# Methemoglobinemia induced by dapsone in a pediatric patient: case report

**DOI:** 10.5935/2965-2774.20230018-en

**Published:** 2023

**Authors:** Ana Clara Burgos, Alexandre Neves da Rocha Santos, José Colleti Junior, Eduardo Juan Troster

**Affiliations:** 1 Faculdade Israelita de Ciências da Saúde Albert Einstein, Hospital Israelita Albert Einstein - São Paulo (SP), Brazil; 2 Hospital Israelita Albert Einstein - São Paulo (SP), Brazil

## INTRODUCTION

Methemoglobinemia is a rare condition and one of the differential diagnoses of cyanosis in the pediatric age group.^(1)^ Clinical symptoms vary according to the levels of methemoglobin (MetHb) in the blood and may be nonspecific. The most common symptoms are central cyanosis, headache, fatigue, and respiratory depression.^(2)^ Therefore, it is essential to recognize and treat the underlying cause. Methemoglobinemia is a syndrome of varied etiology, which may be congenital or acquired. The main acquired cause is a reaction to chemical agents.^(1)^ One of the drugs most commonly associated with methemoglobinemia is dapsone, a sulfone antibiotic. Its traditional indication is for the treatment of dermatitis herpetiformis, but it is also used in the treatment of leprosy and in the prophylaxis of *Pneumocystis jiroveci* and toxoplasmosis.^(2-5)^ Its use in oral form for the treatment of acne vulgaris is not well established.^(6)^

This case report presents a patient treated at a pediatric emergency department and her outcome, aiming to discuss the diagnostic difficulties of methemoglobinemia in pediatrics and to draw the pediatric community’s attention to the potential severity of the diagnosis and the indiscriminate use of dapsone.

This study was approved by the Ethics Committee of the *Hospital Israelita Albert Einstein* (HIAE) upon acceptance of the Free Consent Form and CAAE 65121122.6.0000.0071.

## CASE REPORT

A 15-year-old female patient had been on continuous oral dapsone for the treatment of acne vulgaris for 18 days. She sought the children’s emergency department of a secondary hospital on August 28, 2022, complaining of central and peripheral cyanosis that had been progressively worsening for 2 days. She reported nonspecific symptoms of headache, dizziness and myalgia in the previous week, with spontaneous improvement. In the first consultation, she had a maximum oxygen saturation of 83% on room air, without any complaints of dyspnea or discomfort in the associated respiratory tract. After collection of exams, her MetHb was 9.7% (reference value 0.0 - 1.5), establishing the diagnosis of methemoglobinemia. A dose of 1mg/kg methylene blue was administered intravenously. In a new laboratory examination, she had MetHb 2.2% (no time reported). In a third control, MetHb was found to be 3.4%, so another dose of methylene blue was administered. Hospitalization was requested on the same day for expert clinical care and diagnostic investigation. The patient was transferred to the intensive care unit (ICU) of HIAE. Laboratory test results are presented in table 1.

**Table 1:** Laboratory tests

	External service	*Hospital Israelita Albert Einstein*	Reference values
	27 August 2022	29 August 2022 - 1h45	
Red cells	-	3.45	4.10 - 5.10
Hemoglobin	11.5	10.1	12 - 14.8
Hematocrit	34	30	36 - 43
Leukocytes	6,680	6,200	4,500 - 13,000
Neutrophils	59%	62%	-
Eosinophils	3%	5.2%	-
Basophils	1%	0.3%	-
Lymphocytes	24%	23.4%	-
Monocytes	13%	8.9%	-
Platelets	171,000	193,000	150,000-450,000
Direct bilirubin	-	0.4	0 - 0.3
Indirect bilirubin	-	0.7	0.1 - 0.9
Total bilirubin	-	1.1	0.1 - 1.2
Ionic calcium (mmol/L )	1.17	1.17	1.14 - 1.31
Creatinine	0.7	0.56	0.50 - 0.90
Alkaline phosphatase	-	77	57 - 254
GT Range	-	11	4 - 16
Glucose	81	100	70 - 99
Magnesium	-	1.7	1.3 - 2.1
pH (arterial oximetry)	-	7.374	7.35 - 7.45
Potassium	3.4	3.9	3.5 - 5
Sodium	139	138	135 - 145
TGO	24	15	0 - 23
TGP	16	10	0 - 18
Urea	28	19	18 - 45
pCO_2_	-	42.8	35 - 45
pO_2_ (arterial blood gas analysis)	-	37.8	80 - 90
BE (arterial blood gas analysis)	-	- 0.3	- 2 - 2
HCO_3_ (arterial blood gas analysis)	-	24. 4	24 - 28
Total CO_2_ (arterial blood gas analysis)	-	25. 7	23 - 27
Hb saturation (arterial blood gas analysis)	-	66.1	96 - 97
p50 (arterial blood gas analysis)	-	29.9	24 - 28
FO_2_Hb	-	64.1	94 - 99
Carboxyhemoglobin	-	0.1	0 - 2
Methemoglobin	August 27, 2022: 2.2% (after methylene blue	2.9	0.2 - 0.6
	28 August 2022: 3.4%		
	29 August 2022: 2.9%		

Gamma GT - gamma-glutamyl transferase; TGO - oxaloacetic transaminase; TGP - pyruvate transaminase; pCO_2_ - partial pressure of carbon dioxide; pO_2_ - partial pressure of oxygen; HCO_3_ - bicarbonate; CO_2_ - carbon dioxide; FO_2_Hb - oxyhemoglobin fraction.

On admission to the ICU, the patient was in fair general condition, ruddy, hydrated, acyanotic, anicteric, afebrile, reactive, and cooperative. On examination of the cardiovascular system, her heart rate was 100bpm, and her blood pressure was 105/75mmHg. On cardiovascular auscultation, she had two-beat, normophonetic rhythmic sounds without audible murmurs. The patient complained of mild pain in the precordial region, without radiation.

On pulmonary examination, she presented bilateral breath sounds, without adventitious sounds and with 98% saturation on room air. The capillary refill time was 2 seconds, with no signs of edema. Other systems showed no changes on physical examination. On admission, laboratory tests and electrocardiograms were collected due to chest pain, and dipyrone 500mg was administered orally.

During hospitalization, the patient required oxygen through a nasal catheter at 1L/minute on the first day, becoming asymptomatic and maintaining the MetHb values within the normal range. No more doses of methylene blue were needed. The patient was discharged after 2 days, evolving with complete resolution of symptoms.

## DISCUSSION

Methemoglobinemia and its consequences are well defined. However, this condition is not always considered a differential diagnosis in hospital emergency departments that present with acute cyanosis without signs of associated heart disease.^(1)^

Methemoglobinemia is characterized by MetHb > 2% in the blood.^(1)^ Its increase is due to the oxidation of the iron portion of hemoglobin from the ferrous state (Fe^2+^) to the ferric state (Fe^3+^). This transformation causes hemoglobin to have a lower affinity for oxygen, making it unable to transport it and shifting the dissociation curve of oxygen to the left.^(2)^ The result is tissue hypoxia. Under normal circumstances, low MetHb levels of approximately 1% are maintained through regulatory mechanisms.^(3)^ The cytochrome b5-MetHb reductase pathway is responsible for 95-99% of the reduction and removal of MetHb. The nicotinamide adenine dinucleotide phosphate (NADPH)-dependent pathway requires glucose-6-phosphate dehydrogenase (G6PD) and is responsible for only about 5% of the reduction. This pathway may be potentiated by exogenous factors, such as methylene blue.^(3)^

The diagnosis of methemoglobinemia is suspected in the presence of clinical symptoms associated with low oxyhemoglobin saturation, as evidenced by pulse oximetry and confirmed by arterial blood gases.^(5)^ Although methemoglobinemia is a rare cause of cyanosis in children, its treatment is specific. Therefore, it is important that this hypothesis be considered and investigated by measuring MetHb in the blood, allowing treatment to be started early.

The treatment of methemoglobinemia varies with the severity of the patient’s symptoms. Asymptomatic patients with MetHb levels < 20% need only support and exclusion of the cause of the disorder. For patients with MetHb > 20% or who are symptomatic, the recommendation is immediate treatment with the antidote.^(3)^ Methylene blue is the drug of choice. Its mechanism of action provides the reduction of MetHb in hemoglobin again. However, high doses of vitamin C work as an effective alternative.^(2)^ In general, an intravenous dose of methylene blue (1 - 2mg/kg) for 5 min is sufficient to provide a significant drop in blood MetHb levels, with improvement of symptoms. The need for a second administration is rare.^(2)^ Methylene blue should be given with caution, as its excess can lead to risks for the patient, such as hemolysis and paradoxical increase in MetHb to levels greater than 10%.^(3)^ It is noteworthy that in the patient here, a second dose of methylene blue was administered, even though the patient was asymptomatic and had MetHb = 3.4%, i.e., going against the clinical recommendation. Nevertheless, the patient evolved well and had no bad outcomes.

Most cases of methemoglobinemia induced by dapsone occur under its most common uses and are already strongly established in the literature, such as for cases of leprosy and dermatitis herpetiformis. There is a need to pay attention to other, less frequent uses of dapsone. In the present case, oral dapsone was being used for the treatment of acne vulgaris, which, although correct, is not routinely used in dermatology due to potential topical side effects.^(6,7)^ Pediatricians and dermatologists should be aware of the side effects of long-term use of dapsone.
